# Towards the genetic control of invasive species

**DOI:** 10.1007/s10530-017-1384-6

**Published:** 2017-02-21

**Authors:** Tim Harvey-Samuel, Thomas Ant, Luke Alphey

**Affiliations:** 1 The Pirbright Institute, Pirbright, UK; 2 University of Glasgow, Glasgow, UK

**Keywords:** Invasive species, Biodiversity conservation, Genetic pest management, Genetic control, Transgenic control, Refractory transgene, Eradication

## Abstract

Invasive species remain one of the greatest threats to global biodiversity. Their control would be enhanced through the development of more effective and sustainable pest management strategies. Recently, a novel form of genetic pest management (GPM) has been developed in which the mating behaviour of insect pests is exploited to introduce genetically engineered DNA sequences into wild conspecific populations. These ‘transgenes’ work in one or more ways to reduce the damage caused by a particular pest, for example reducing its density, or its ability to vector disease. Although currently being developed for use against economically important insect pests, these technologies would be highly appropriate for application against invasive species that threaten biodiversity. Importantly, these technologies have begun to advance in scope beyond insects to vertebrates, which include some of the world’s worst invasives. Here we review the current state of this rapidly progressing field and, using an established set of eradication criteria, discuss the characteristics which make GPM technologies suitable for application against invasive pests.

**Electronic supplementary material:**

The online version of this article (doi:10.1007/s10530-017-1384-6) contains supplementary material, which is available to authorized users.

## Introduction

Invasive species are a “leading cause of animal extinctions” worldwide (Clavero and Garcia-Berthou 2005) and represent one of the greatest threats to global biodiversity (IUCN 1999). Current methods for their control are in many cases inadequate to prevent continuing biodiversity loss (Genovesi 2011; Thresher 2007; Bax and Thresher 2009). Toxins/pesticides, classical biological control, trapping/hunting, and habitat removal may only be suitable for certain taxonomic groups or species (Myers et al. 2000; Courchamp et al. 2003). Moreover, their deployment is often expensive and labour-intensive (Bomford and O’Brien 1995; Simberloff 2009), can be associated with significant off-target effects (Courchamp et al. 2003; Pitt et al. 2015; Zarnetske 2010), and may, more realistically, be aimed at continued management rather than eradication (Simberloff 2009; Myers et al. 1998). With the rate of invasions both unprecedented and increasing (Pimentel et al. 2005), there is a serious need for the development of alternative, effective and sustainable methods for invasive species control (Glen et al. 2013; Thresher et al. 2014; Brockerhoff et al. 2010).

A form of pest control that is potentially highly suitable for combatting invasive species is known as genetic pest management (GPM). GPM is a form of biological control that exploits a pest’s mate-seeking behaviour to introduce harm-reducing genetic modifications into wild pest populations (Curtis 1985). In GPM a pest is colonized, modified to carry one or more heritable control traits and then released into a target population where it will pass these modifications to its offspring. A GPM strategy that has been in use for decades in agriculture is the sterile insect technique (SIT) (Klassen and Curtis 2005) which uses radiation to induce sterility in insect pests prior to release. Since wild females mating a released sterile male have few or no viable offspring, mass-release of radiation-sterilised pests reduces the reproductive potential of the target population and over time can suppress or even eradicate it.

Recently, a range of novel, transgene-based, GPM strategies have been developed (Burt 2014; Alphey 2014) that have the potential to widen the scope of GPM to a variety of intractable invasive pests (Gould 2008). Here the heritable modifications transported into wild populations are synthetic DNA sequences (‘transgenes’) engineered into the pest genome. These ‘next generation’ GPM technologies offer significant advantages over current methods such as the SIT in that they can be more efficient and provide greater flexibility in the control traits they induce. Moreover, they are applicable across a broader taxonomic range and allow engineering/re-engineering by design (Black et al. 2011).

**Fig. 1:**
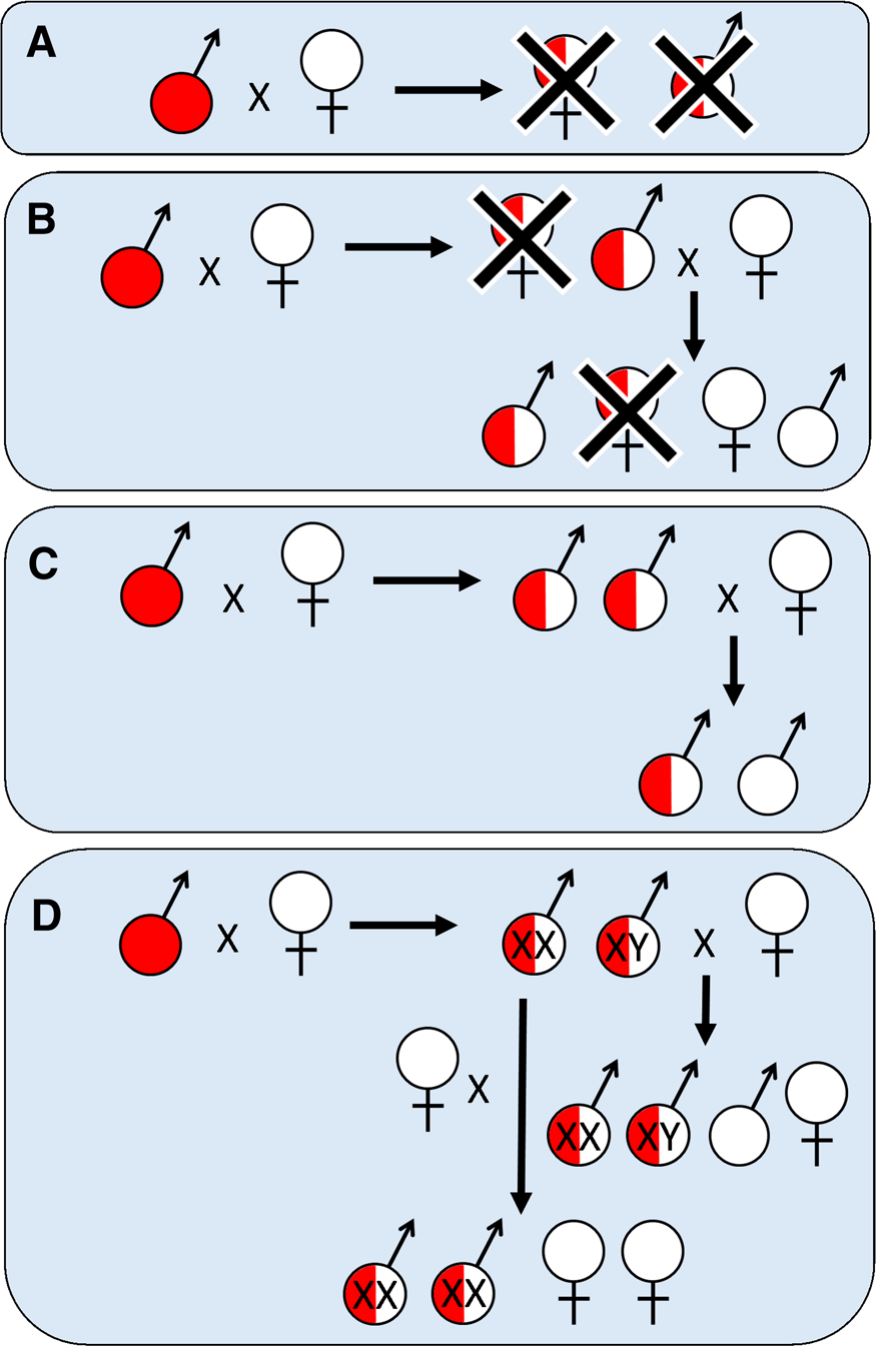
Inheritance and effects of self-limiting population suppression strategies once released into target populations. Released individuals are transgene homozygous males (*full red circle*) which mate with wild-type females (*full white circle*), producing F_1_ heterozygotes (*red*/*white semicircle*). **A** Bisex-lethal transgene: All F_1_ individuals die. **B** Female-lethal: F_1_ females die. F_1_ males survive and pass on the transgene to 50% of their F_2_ progeny; female F_2_ transgene carriers also die. **C** Nuclease-based sex ratio distortion (SRD)—described in main text using HEGs. Nuclease expressed from transgene located on autosome. X-chromosomes are destroyed by action of the nuclease during meiosis so that transgenic males produce only Y-bearing sperm. All F_1_ progeny are male and are transgene heterozygotes. Heterozygous males will pass the transgene to their (all male) F_2_ progeny at Mendelian ratios. Note that the same design but with the nuclease transgene located on the Y-chromosome instead of an autosome provides a self-sustaining suppression system (described in main text as Y-drive). Hemizygous males then produce exclusively male offspring—all of which carry the Y-located transgene. **D** Aromatase SRD: All F_1_ progeny will be phenotypically male, however 50% will carry female sex chromosomes (XX) “pseudomales”. In the F_2_ generation, 75% of progeny produced by heterozygous XY males will develop as phenotypic males (XX heterozygotes converted to pseudomales) while XX pseudomales will produce progeny at a 50:50 phenotypic sex ratio

**Fig. 2:**
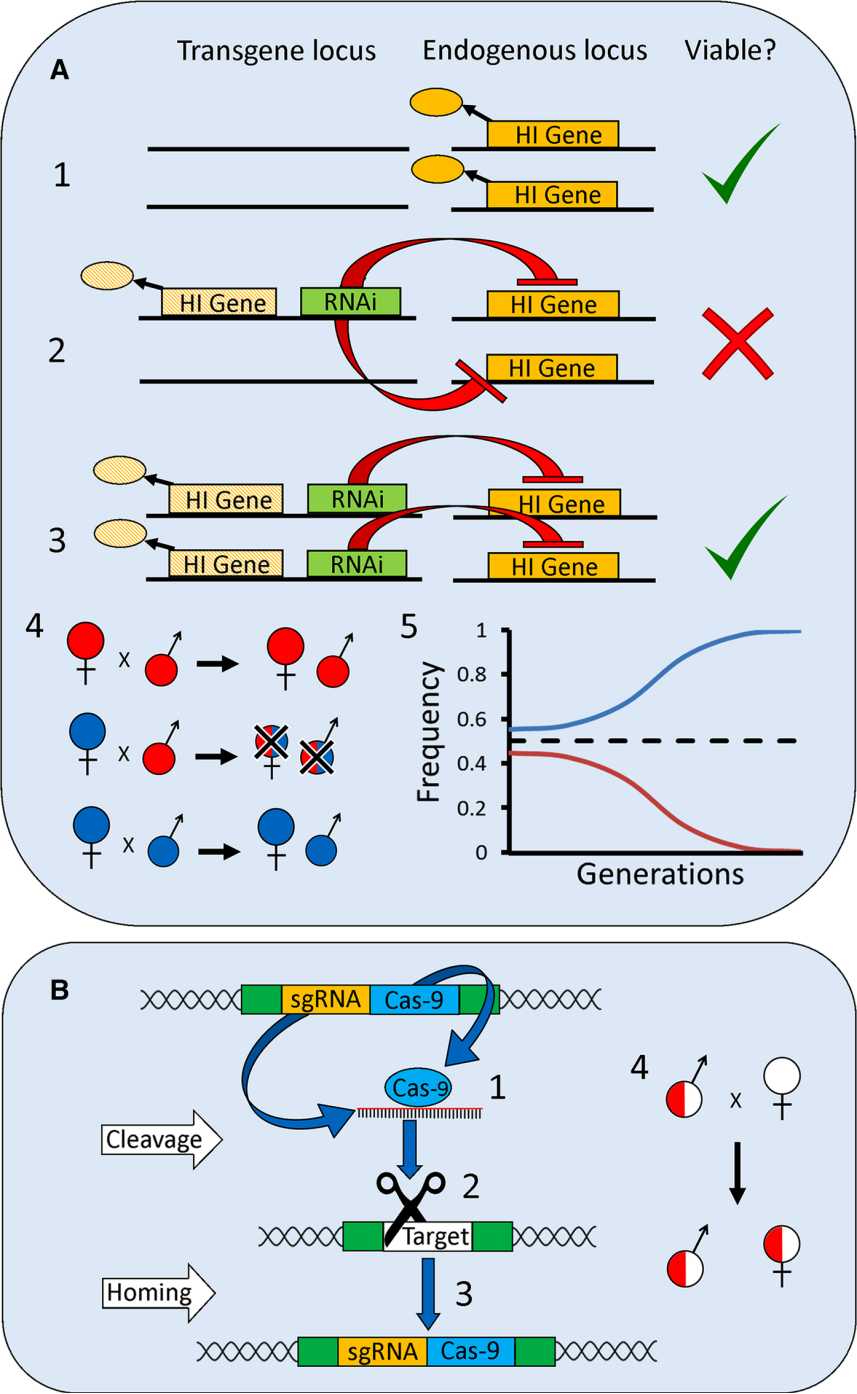
Inheritance and molecular mechanisms of two self-sustaining (gene-drive) strategies. **A** engineered haploinsufficient underdominance: Shown is a generic single-locus system analogous in function to Reeves (2014). In this system all individuals in the target population are homozygous wildtype at the *endogenous* haploinsufficient locus (HI gene—*solid orange*). Three genotypes are possible at the *transgene* locus. (*1*) Wild-type carrying no copies of the transgene (viable). (*2*) Transgene heterozygotes with one copy of the *RNAi*-*resistant* ‘rescue’ Haploinsufficient Gene (HI gene—*orange hatched*). This genotype has only one functional copy of the HI gene—the single transgenic copy, the two endogenous copies being suppressed by RNAi—and therefore suffers reduced fitness (non-viable). (*3*) Transgene homozygotes where *endogenous* HI gene expression is also disrupted, but is rescued by the presence of two copies of the *RNAi*-*resistant* HI gene (viable). (*4*) Once released into a target population, individuals will therefore suffer from reduced fitness if they mate with another genotype (heterozygotes have low fitness). (*5*) This creates an unstable equilibrium with the more predominant allele being driven to fixation. In the absence of transgene fitness costs, the invasion threshold of this system is 0.5 (*black dotted line*). **B** CRISPR–Cas9 homing-drive: depicted individual is initially transgene heterozygous. Homologous genomic regions are represented in *green*. (*1*) Cas9 endonuclease is expressed from the transgene and directed to a target site complementary to the linked and co-expressed sgRNA sequence. This sgRNA target site occurs within a genomic region homologous to the transgene insertion site (e.g. a wild-type homologous chromosome). (*2*) Cas9 cleaves this target locus and due to the homology of the transgene’s flanking regions with the cut target site it is used as a template for homology-directed DNA repair (HDR). (*3*) During this process the transgene is copied (homes) into the cleaved target locus creating a transgene homozygous cell. (*4*) If this process occurs in the germline, offspring of individuals heterozygous for the sgRNA-Cas9 transgene will inherit the gene-drive at above normal rates (‘super-Mendelian inheritance’). For example, transmission rates above 90% have been observed in *Drosophila* and mosquitoes (Hammond et al. 2016; Gantz 2015; Gantz and Bier 2015), rather than the 50% expected from Mendelian inheritance

Depending on the design of the transgene, these GPM strategies can have different effects on their target pest population. *Population suppression* strategies aim to reduce pest densities or eradicate them completely, for example removing an invasive rat population from a Pacific island. Alternatively, *population replacement* strategies aim to modify a wild pest in order to eliminate an undesirable effect, without necessarily altering its population size. Such a strategy could involve reducing the vectorial capacity of a wild mosquito population through the release of individuals carrying a pathogen-blocking transgene (see “Box 1: Disease-refractory transgenes” section). More fundamentally, both population suppression and replacement strategies may vary in the extent to which their transgenes persist or spread in a target population. In *self*-*limiting* strategies (Fig. 1), transgenes are expected to disappear from a wild population unless maintained by the periodic release of additional engineered pests. In a sense, this approach is similar to biopesticides that may require multiple applications against a target population. Conversely, in *self*-*sustaining* strategies (Fig. 2), transgenes are expected to persist indefinitely, potentially increasing in allele frequency within the target population and/or spreading to populations beyond the initial release area (Alphey 2014).

Currently, GPM strategies are being developed primarily to combat insect pests of human health or agricultural economic importance. However, there is increasing interest in using these technologies as conservation tools for the control of invasive species that threaten biodiversity (Thresher et al. 2014; Gould 2008; Simberloff 2014; Campbell et al. 2015) (see “Box 2: Case studies” section). As GPM transgene designs and components have already been transferred between closely related insects (see “Transferability” section) (Jin et al. 2013; Tan et al. 2013; Ant et al. 2012; Leftwich 2014; Fu et al. 2010; Marinotti et al. 2013), it is likely that they could be applied to other invasive pests within this Class with relatively minor adjustments. Moreover, a number of these technologies are now at various stages of development in vertebrates (Grewe 2007; Thresher et al. 2000, 2009, 2014) (D. Threadgill: personal communication), which include amongst the world’s most harmful invasive species (Lowe 2000).

Comprehensive descriptions of the various GPM designs that have been proposed are available elsewhere (Burt 2014; Alphey 2014). Here we review the state-of-the art in transgene-based GPM strategies targeting insects and vertebrates, with emphasis on those technologies being developed for invasive pests. We provide an overview of the genetic mechanisms involved and explore the characteristics that may make GPM strategies suitable for eradicating invasive populations. Additionally, we discuss the primary concern surrounding the deployment of self-sustaining strategies (transgene escape) and proposed means for mitigating this concern. Finally, through the use of case studies, we consider the potential for GPM to be deployed against currently intractable invasive species from a range of taxa.

## Self-limiting strategies

At the core of self-limiting GPM strategies is the transient introduction of transgenes into a population which would return to its unmodified state were releases to cease. Periodic release of additional transgenic individuals is therefore required to maintain the control effect (suppression or replacement). Generally, it is thought that population replacement strategies will require the deployment of gene-drive systems (see “Self-sustaining strategies” section) for disease-refractory transgenes to be maintained in wild populations at frequencies high enough to substantially reduce transmission rates (Sinkins and Gould 2006) (but see Rasgon 2009; Gould et al. 2008). We therefore limit discussion in this section to strategies aimed at population suppression.

Broadly, self-limiting *population suppression* strategies involve transgenes which either cause lethality to pre-reproductive stages (Fig. 1A, B), or which skew the population sex ratio in favour of males (Fig. 1C, D). Both act to reduce the reproductive potential of the target population, primarily by reducing the number of females. Once released, persistence of these transgenes in target populations will be limited by fitness costs associated with transgenesis and laboratory adaptation (reviewed in Marrelli et al. 2006), compounded in lethal systems by the engineered control phenotype (death) (Harvey-Samuel et al. 2014). These costs can be estimated in both the laboratory (Harvey-Samuel et al. 2014) and field (Harris et al. 2011), allowing for the calculation of relative fitness parameters and persistence times of self-limiting transgenes in mixed populations. A benefit of this limited persistence is that the deployment of self-limiting systems is inherently reversible: if unintended consequences were to arise from a release programme, the target population could be allowed to return to its unmodified state simply through the cessation of releases (Gould 2008; Rasgon 2009).

### Self-limiting population suppression strategies

#### Bisex-lethal systems

A bisex-lethal transgene causes mortality to both males and females inheriting it in the field (Fig. 1A). The most developed of these systems utilizes the ‘RIDL’ (release of insects carrying a dominant lethal genetic system) technology (Thomas et al. 2000). RIDL insects carry a transgene with a dominant lethal phenotype. RIDL lethality can be suppressed prior to field release by providing insects with an antidote (tetracycline or suitable analogues), usually in their larval diet. In the absence of sufficient quantities of tetracycline in the field (Curtis 2015), progeny inheriting RIDL transgenes from released insects die before reproducing. As with the SIT, periodic releases of RIDL insects are therefore required to maintain a population of transgenic insects in the field in order to continue to suppress the target population. However, RIDL insects have no need to be irradiated prior to field release, a process which can significantly impair SIT programme efficacy through reducing insect mating competitiveness (Calkins and Parker 2005).

Functioning bisex-lethal strains have been developed in a range of economically important agricultural pests such as the pink bollworm *Pectinophora gossypiella* (Morrison 2012), Mediterranean fruit fly (medfly) *Ceratitis capitata* (Gong et al. 2005) and the dengue fever mosquito *Aedes aegypti* (Phuc et al. 2007). Open field suppression trials releasing males from this RIDL mosquito line in Grand Cayman (Harris et al. 2012) and Itaberaba, Brazil (Carvalho 2015) resulted in 85 and 95% suppression of the wild target population, respectively. Both trial sites were contiguous with untreated infested areas from which wild-type insects could disperse, therefore 100% suppression, i.e. local eradication, was not possible or anticipated in these small trials.

Research towards developing tetracycline-suppressible bisex-lethal strategies in vertebrates (rodents and fish) and a mollusc has been conducted to the proof-of-principle stage (Grewe 2007; Thresher et al. 2009). Results were particularly encouraging in the Channel catfish (*Ictalurus punctatus*), where integrated transgenic lines over-expressing a dorsalising gene (*BMP2*) showed 95.6% mortality which could be repressed to wild-type control levels through provision of a tetracycline analogue (doxycycline) (Thresher et al. 2014). Transient assays in the Pacific oyster (*Crassostrea gigas*) have also shown success with transgenes engineered to knockdown a homeobox gene (*HOXCG1*) resulting in 67% larval mortality, which again could be repressed to control levels through the provision of doxycycline. Designed as biocontainment systems for domesticated stocks, these technologies act as a form of preventative invasive species control by reducing the likelihood of farmed species establishing in non-native areas. Moreover, if transferred into related invasive pests and released into wild populations they could function as suppression systems (Thresher 2007; Thresher et al. 2014).

#### Female-lethal systems

Transgenic systems which cause lethality only in females can be more efficient at population suppression than bisex-lethal designs, particularly if engineered into multiple independently segregating loci (Bax and Thresher 2009; Schliekelman et al. 2005; Schliekelman and Gould 2000). This increased efficiency derives from the viability of male transgene heterozygotes which disseminate the transgene in subsequent generations (see Fig. 1b). Additionally, this approach facilitates sex separation during rearing through induction of the lethal phenotype in females prior to release (Papathanos 2009). Male-only releases can dramatically improve suppression efficiency (e.g. 3–5× in medfly) by concentrating the mate-seeking and reproductive behaviour of released individuals on target population females (Hendrichs et al. 1995; Rendon et al. 2004).

Female-specific RIDL (fsRIDL) systems have been developed by combining deleterious effector genes with female-specific gene regulatory elements, for example female-specific promoter/enhancers (Thomas et al. 2000; Heinrich and Scott 2000) or alternatively spliced sex-specific introns (Jin et al. 2013; Fu et al. 2007, 2010), often derived from genes involved in the sex-determination cascade (Dafa’alla et al. 2010). fsRIDL lines have been generated in a variety of Lepidoptera (Jin et al. 2013; Tan et al. 2013), tephritid fruitflies (Ant et al. 2012; Fu et al. 2007; Schetelig and Handler 2012; Morrison et al. 2010), calliphorid blowflies (Yan and Scott 2015; Concha 2016) and mosquitoes (Fu et al. 2010; Marinotti et al. 2013; Labbe 2012) with preliminary research now extending this technology to the Coleoptera (Gregory 2015). Repeated releases of fsRIDL transgenic insects have shown the capacity to rapidly eliminate conspecific caged populations of *Ae. aegypti* (de Valdez et al. 2011) and agricultural pests including the diamondback moth (*Plutella xylostella*), medfly and olive fruit fly (*Bactrocera oleae*) (Ant et al. 2012; Leftwich 2014; Harvey-Samuel 2015). With germ-line transformation now possible in a variety of insect Orders (Gregory 2016) and increasing understanding of insect sex determination cascades (Verhulst et al. 2010), it is probable that bisex-lethal and female-specific RIDL strategies could be transferred to a number of invasive insects that threaten biodiversity (see “Box 2: Case studies -Insects” section).

A tetracycline repressible female-lethal system has been designed for invasive fish and demonstrated in the zebrafish (*Danio rerio*) and common carp (*Cyprinus carpio*) (Thresher et al. 2014). A zebrafish line carrying this transgene showed high female mortality when reared off-tetracycline (up to 100% males), whereas on-tetracycline sex ratios fell within normal wild-type levels. Spatiotemporal transgene expression in this system is under the control of the promoter region from *vitellogenin 1*: a gene expressed during egg production. As *vitellogenin 1* is ubiquitous amongst oviparous vertebrates it is argued that this system would be transferrable to other egg-laying invasives such as the brown tree snake (*Boiga irregularis*), cane toad (*Bufo marinus*) and Nile perch (*Lates niloticus*). Modelling suggests that use of female-lethal methods could be effective at eradicating a range of invasive vertebrates (Thresher et al. 2014; Thresher 2007; Bax and Thresher 2009).

#### Sex-ratio distortion systems

In female-lethal systems, the transgene allele frequency is halved each generation as female carriers are not viable. Alternatively, in a sex-ratio distortion (SRD) system, the progeny of transgenic individuals are viable but develop as a single sex (usually male). SRD systems cause suppression through biasing the sex ratio of a target population in favour of males, with a corresponding reduction in females. Designs for self-sustaining SRD systems exist but will be discussed separately (see “Homing-based drives” section). SRD systems are predicted to be substantially more efficient at population suppression than female-lethal strategies (Schliekelman et al. 2005).

A self-limiting SRD strategy has been developed in insects using homing endonuclease genes (HEGs) (Galizi et al. 2014). HEGs are naturally occurring selfish genetic elements that replicate in a host genome by expressing an endonuclease that can bind and cleave a specific 20–30 bp DNA sequence (Deredec et al. 2008). It was observed that binding/cleavage sites for I-PpoI (a HEG from the slime mould *Physarum polychephalum*) occur in multiple copies in the genome of the human malaria vector *Anopheles gambiae* and, fortuitously, that these loci are exclusively located on the X-chromosome. When I-PpoI endonuclease was expressed during spermatogenesis in male transgene carriers, a high proportion of X-chromosomes were destroyed, strongly biasing transmission of Y-chromosome-bearing sperm during mating (up to 97% male progeny) (Fig. 1c).

A SRD system designed for application against invasive fish has been developed which results in phenotypic masculinization of genotypic females through RNAi knockdown of a hormone (aromatase) required for female sexual development (Thresher 2005). Transgene carriers develop as males regardless of their sex chromosomes, remain fertile and can successfully mate with wild-type females (Fig. 1d). Aromatase SRD transgenes have shown functionality in transient assays of zebrafish, Medaka (*Oryzias latipes*) and common carp, with fitness and efficacy trials of transgenic carp ongoing (R. Thresher: personal communication). Aromatase serves a key function in female sex-determination in many lower vertebrates and when chemically blocked can cause sex-reversal in female birds, amphibians, reptiles and fish. It may therefore be useful in the development of SRD strategies for a variety of invasive vertebrates (Thresher 2007) (see “Box 2: Case studies - Reptiles” section).

Preliminary research has been conducted into developing a SRD system for control of the cane toad in Australia, with candidate primary sex determination genes now identified (Koopman 2006; Abramyan et al. 2009a, b), and has been proposed for the control of invasive bighead carps (*Hypophthalmichthys* spp.) and parasitic sea lampreys (*Petromyzon marinusin*) in North America (Asian-Carp-Working-Group 2007; Bergstedt and Twohey 2007).

## Self-sustaining strategies

Under some circumstances, the frequent releases typically required by self-limiting systems may be ecologically inappropriate or cost prohibitive. This could be the case where the adult release stage is itself damaging (e.g. some coleopteran and mammalian pests) or where the target species is difficult or expensive to rear in large numbers. Additionally, it may be difficult to sustain transgene frequencies at high levels using self-limiting strategies: a potential issue where the goal is to spread disease-refractoriness (Boete and Koella 2003).

Self-sustaining strategies overcome these issues through engineering transgenes that persist in and spread through the population into which they are introduced. The control phenotype is therefore disseminated through a pest population by the autonomous ‘selfish’ behaviour of the transgene itself. All self-sustaining strategies are based on synthetic, selfish genetic elements (‘gene-drive systems’) that bias reproductive outcomes in their favour such that they are preferentially inherited and spread (drive) through a population (Sinkins and Gould 2006). This allows them to increase in frequency without providing a fitness benefit to individual carriers and, potentially, even while being deleterious to such carriers.

If a gene expressing a particular control phenotype is closely linked to a gene-drive system, this ‘cargo’ gene will be inherited and spread through a target population along with the gene-drive. The outcome of a self-sustaining strategy (suppression or replacement) is therefore dependent on the phenotype of its linked cargo gene. It is also possible to have an effect without a cargo gene, instead using the gene-drive insertion to knock out or modify the endogenous target sequence into which it inserts. This is mainly relevant to homing drives (see “Homing-based drives” section). A variety of synthetic gene-drive systems have been proposed (Burt 2014; Sinkins and Gould 2006). Here we focus on engineered haploinsufficient underdominance (Reeves 2014) and homing-based drives built using the CRISPR–Cas9 nuclease system (Jinek et al. 2012; Esvelt 2014).

### Engineered haploinsufficient underdominance

Underdominance describes a scenario where a heterozygote is less fit than either parental homozygote, the opposite of the well-known phenomenon of ‘hybrid vigour’. Assuming equal fitness of two competing underdominant alleles, an unstable equilibrium is created, with the most common allele tending towards fixation and the other towards extinction (see Fig. 2A). This behaviour has long been recognized as a potential means of spreading genetic traits (Curtis 1968), including transgenes (Davis et al. 2001), through a pest population.

Recently, a transgene-based underdominance system was engineered in *Drosophila melanogaster* utilizing the *Minute* gene, *RpL14* (Reeves 2014). *RpL14* displays haploinsufficiency: two copies of the gene (either as alleles at the same locus, or independent copies at two loci) are required for full fitness. The underdominant transgene contained an RNAi component designed to disrupt expression of endogenous *RpL14*, while simultaneously expressing a synthetic RNAi-resistant ‘rescue’ version of the same gene (see Fig. 2A). Wild-type and transgene-homozygous individuals each possessed two functional copies of *RpL14.* Transgene heterozygotes, however, had the function of both endogenous *RpL14* copies reduced by RNAi with only a single *RpL14* rescue copy on the transgene and consequently displayed strongly reduced fitness. Release of insects carrying the underdominant transgene into caged populations above the unstable equilibrium frequency resulted in the transgene increasing to fixation.

*RpL14* is one of the cytoplasmic ribosomal protein genes. These genes show broad phylogenetic conservation and their haploinsufficiency has been characterised in fungi, plants, invertebrates and vertebrates (Kim et al. 2010; Marygold 2007; Perry 2007). They may therefore provide the basis for underdominance-based gene-drive systems in a variety of invasive species (Reeves 2014). Although still at the prototype stage, the *RpL14* system is being developed as a potential mechanism for spreading avian malaria (*Plasmodium relictum*) refractory transgenes into invasive populations of *Culex quinquefasciatus* on Hawaii (F. Reed: personal communication—see “Box 1: Disease-refractory transgenes” section). Due to the reduced viability of heterozygotes, underdominance systems can theoretically cause suppression (Serebrovskii 1940) and have been used to eradicate caged mosquito populations (Laven et al. 1972). However, current research is focused on developing them for population replacement strategies and as a means of increasing the confinement of other gene-drive systems (see “Transgene escape” section).

### Homing-based drives

A cell may attempt to repair a double-stranded DNA break either by joining the cut ends together, known as ‘non-homologous end-joining’ (NHEJ), or by using the ‘homology-directed repair’ (HDR) pathway which uses undamaged homologous DNA sequences as a repair template. Homing-based gene-drives (homing-drives) increase their frequency through cleaving DNA at specific genomic locations and then being copied into the cleavage site through HDR (a process known as ‘homing’) (Fig. 2B). Homing thus requires the homing-drive transgene to be located within a region showing sequence homology to its cleavage site. If homing occurs at a high enough frequency in the germ-line to counteract fitness costs imposed by the gene-drive, it can result in greater than Mendelian inheritance of the transgene and its spread through a population (Burt 2003).

The first demonstration of homing-drive systems used HEGs (Windbichler et al. 2011). CRISPR–Cas9 nucleases now provide more flexible tools for this purpose (Jinek et al. 2012; Esvelt 2014). Similar to HEGs, Cas9 genes express an endonuclease which binds to and cleaves specific DNA sequences. However, these target cleavage loci are defined not by the DNA binding affinity of the endonuclease (as in HEGs), which may require laborious and complex protein engineering techniques to respecify, but by complementary single-guide RNAs (sgRNAs). sgRNA coding sequences can be linked to the Cas9 gene allowing the endonuclease and targeting system to home as a single unit (Fig. 2B). The ease with which sgRNAs may be reengineered (and therefore different genomic sequences targeted) constitutes a major advance in broadening the potential genes and species against which homing-drives may be applied (Esvelt 2014).

Homing-drives have been proposed both as a mechanism for spreading closely linked disease-refractory transgenes through target populations (Burt 2014) and as self-sustaining suppression systems (Deredec et al. 2008), primarily in mosquitoes but in principle also other taxa, e.g. the invasive weed Scotch broom *Cytisus scoparius* (Gould 2008). For suppression, fitness costs could be imposed through engineering the drive to home into and disrupt the function of an important endogenous gene (insertional mutagenesis) thereby imposing a fitness load on the target population as it spreads. Such a suppression drive was recently demonstrated in the human malaria mosquito *An. gambiae* (Hammond et al. 2016). CRISPR/Cas9 drive transgenes were directed to target genes required for female fertility. These homing drives displayed three characteristics required for efficient suppression of a population; (1) their phenotype was not dominant, allowing spread of the drive at low-medium allele frequencies where most carriers are heterozygotes (Burt 2003); (2) their phenotype was female-specific allowing males to continue spreading the drive regardless of their genotype and (3) homing was restricted to the germ-line, ensuring that heterozygous females, while producing predominantly transgenic gametes, were not themselves sterile (Deredec et al. 2008). With homing rates of up to 99%, one of these drive transgenes was able to successfully invade caged populations of *An. gambiae* and was assessed as exhibiting “the minimum requirement for a gene-drive targeting female reproduction in an insect population” (Hammond et al. 2016).

An alternative self-sustaining population suppression strategy using HEG or CRISPR–Cas9 technologies does not rely on homing. Here the transgene is engineered to target and cleave the X-chromosome during meiosis (as in self-limiting HEG-based SRD systems) but is located on the male-specific Y-chromosome. The damaged X-chromosomes are excluded from the mature sperm population creating a meiotic-drive system in which carrier males produce mostly Y-bearing sperm. Individuals inheriting a copy of such a ‘Y-drive’ transgene would therefore be male—and would all carry the drive construct (Hamilton 1967). This is in contrast to self-limiting HEG-based SRD systems where the progeny of transgene carriers are also male, but only 50% are transgenic (no drive). Modelling suggests that release of a Y-drive transgene into a pest population would cause a spreading sex-ratio bias leading to eradication (Deredec et al. 2008). Although not yet demonstrated, Y-drives have been proposed for the control of insects and invasive vertebrate pests (Grewe 2007; Burt 2003).

Both self-sustaining population suppression (Hammond et al. 2016) and replacement (Gantz 2015) transgenes have recently been demonstrated to the proof-of-principle stage in mosquitoes using CRISPR–Cas9 homing-drives. For these strategies to be successfully deployed in the wild, however, numerous issues remain, including technical ones around genetic robustness and stability as well as regulatory and social issues (Alphey 2016). CRISPR–Cas9 technology has shown functionality in invertebrates, plants and vertebrates (including some of invasive importance e.g. Ni 2014; Shao et al. 2014; Square et al. 2015; Perry and Henry 2015), so could likely provide a basis for GPM strategies in a range of invasive species.

### The invasiveness of gene-drives

Once released, gene-drive systems differ in their propensity to spread within, or beyond, a target population—termed ‘invasiveness’. Invasiveness is determined by the invasion threshold: the population allele frequency above which a gene-drive will tend to increase and below which it will tend to decrease. Invasion thresholds vary depending on the mechanics of the drive system and increase with the fitness costs it places on carriers.

Highly invasive gene-drives display zero (no) or low invasion thresholds. Homing-drives can fall within this category and, in general, substantial fitness costs are required to prevent their spread (Marshall and Hay 2012). In population suppression designs, however, invasiveness is highly dependent on the spatial/temporal restriction of homing activity and the dominance of the deleterious control phenotype (Deredec et al. 2008). Underdominance-based systems are generally much less invasive. For the haploinsufficient example described above, the invasion threshold equates to the unstable equilibrium frequency (0.5 in the absence of fitness costs). Beyond these two systems, a variety of other gene-drive technologies/designs exist which display alternative invasion thresholds (Alphey 2014). A caveat of these predicted thresholds is that they are generated using deterministic models. In reality, released transgenes will be subject to genetic drift, creating stochasticity around each theoretical invasion threshold.

#### Transgene escape

Perhaps the greatest concern surrounding the deployment of gene-drive systems in the field is their potential lack of control: once a drive surpasses its minimum invasion threshold it will spread autonomously. This is advantageous in maximising the effect on a pest population for a given release effort but, concomitantly, spatial confinement of a drive or reversal of its spread in the event of accidental release or unintended effects may present a significant challenge. Concerns relating to invasive species include the spread of a gene-drive system to conspecific populations outside of the target geographic area (including to the invasive species’ native range), and the movement of a transgene into the gene pool of a native non-target species through interbreeding—both of which could have unintended ecological consequences. The consequences of this ‘transgene escape’ will depend on interactions between the technology used, the biology of the target species and the ecological context of deployment. Discussion of these various permutations has been explored for insects using a generalised framework elsewhere (David et al. 2013) and is beyond the scope of this review. Here we review proposed mechanisms for limiting the likelihood of transgene escape in the first instance, or reducing the impact of an escaped drive by halting its spread.

For some systems, such as engineered underdominance, high invasion thresholds can act as a built-in transgene escape prevention measure as they require relatively large levels of immigration (Marshall and Hay 2012) or fertile hybridisation to begin spreading autonomously. However, for highly invasive drive systems (e.g. homing-drives) the invasion threshold may be close to zero. Deployment in relatively isolated island ecosystems may provide some limited level of geographic insulation, dependent on the dispersal capacity of the target species. However, the presence of multiple safeguards designed to mitigate the risk of transgene escape are likely to be a prerequisite for the use of low-threshold gene-drives (Oye et al. 2014).

A number of designs for built-in molecular safeguards have emerged aimed at enabling controllable and species-specific gene-drive systems. A proposed method for building a constrained low-threshold gene-drive uses a linear series of independent CRISPR–Cas9 elements configured so that no individual element is autonomous, but depends on the element immediately below it in the series for drive (Noble 2016). The top element in the so-called ‘daisy-chain’ drive carries the payload—e.g. the suppressing transgene. Because the bottom element has no driver (it only drives the element above), it is lost over time through purifying selection. This in turn causes the new bottom element to lose drive capacity, itself becoming susceptible to selection, and so on until all elements including the payload are lost. The top element of a released daisy-chain drive will thus increase to a maximum population allele frequency (increasing with the number of elements in the chain) but, if it fails to reach fixation, will then decrease over time as selection erodes away each basal layer of the chain. This constrains the drive not only temporally but also geographically and taxonomically as multiple elements must escape at relatively high frequencies for a drive to spread substantially in a non-target population.

Further proposals involve post hoc or ‘responsive’ approaches aimed at the neutralisation or alteration of existing gene-drives. So-called ‘reversal’ drives built on the CRISPR–Cas9 architecture can be designed with sgRNA sequences directed to disrupt or alter sequences of an earlier wayward gene-drive (Esvelt 2014), a system which has been demonstrated in laboratory yeast cultures (DiCarlo et al. 2015). These drives overwrite, rather than reverse, the initial drive-induced change, but may in the process be able to revert phenotypic effects of the initial drive. A related approach involves the ‘immunisation’ of non-target populations using drives that recode sequences targeted by the unwanted drive (Esvelt 2014). Immunisation and reversal systems could be combined, with an ‘immunising reversal’ drive having the capacity to spread in both wild-type populations and those altered by a previous drive, simultaneously making non-target populations resistant and neutralising phenotypic effects of the earlier drive (Esvelt 2014). Another design that has been shown to be functional in laboratory populations of *D. melanogaster* is the release of transgenes carrying sgRNA sequences designed to direct Cas9 to cleave its own coding sequence, effectively applying a gene-drive ‘brake’ that is genomically inert until activated by the Cas9 gene from a co-inherited wayward drive (Wu et al. 2016). This latter proposal may prove useful if deployed at sufficiently high frequencies in areas where an escaped drive is expected to spread, though further analysis is required to define the situations, if any, in which this approach may be effective.

Risks associated with gene-drive migration into non-target species can be reduced through the use of target-site sequences unique to the species or sub-population of concern. If such unique sequences are available, a ‘precision drive’ system could be constructed that can only spread in a target population (Esvelt 2014). Alternatively, in the absence of such a naturally occurring sequence a high-threshold gene-drive (e.g. an underdominance system) could first be used to seed a pest population with such a target site (Esvelt 2014). If subsequent checks confirm that the implanted sequence has not spread to non-target populations, a second payload-delivering drive targeting the implanted sequence could follow. As with overwriting drives, precision drives have been demonstrated to the proof-of-principle stage in yeast (DiCarlo et al. 2015). A related approach that could provide very fine spatial control, and may therefore be particularly suited to invasive species management, is the driving of a gene conferring toxicity to a normally benign molecule (Esvelt 2014). Such ‘sensitisation drives’ could, for example, swap a conserved gene required for viability for a version inhibited by a particular molecule. As these genes would have no effect in the absence of the molecule, and the molecule would have no effect in the absence of the gene, target populations in specific areas could be controlled in a manner analogous to the spraying of a highly discriminating pesticide.

## Suitability of GPM for eradicating invasive species

An accepted set of essential requirements for successful invasive species eradication include (1) the control technique must be able to reduce the target species at a rate faster than they are able to reproduce, at all population densities; (2) immigration into the target population must be prevented and (3) resistance to the control technique must not develop (Bomford and O’Brien 1995). An additional desirable feature of a control technology is that it is transferable between target species but remains species-specific when deployed. The general benefits and constraints of GPM strategies have been discussed elsewhere (Thresher et al. 2014; Alphey et al. 2010; Dyck 2005). Here we focus on the suitability of GPM strategies in fulfilling the above criteria.

### Effective across all population densities

This criterion can be broken down into two considerations. Firstly, the control tactic must *remain* effective when population densities are low—for example an incipient population or towards the end of a successful suppression programme. This is a particular weakness of traditional tactics such as pesticides or trapping/hunting as their efficacy declines with population density (Bomford and O’Brien 1995; Klassen 2005) potentially making removal of the last remaining invasive individuals, even over relatively small geographic scales, extremely difficult (Russell et al. 2005). Secondly, the tactic must be able to overcome demographic responses of the target population to suppression. Specifically in the context of invasive species control, the importance of accounting for negative and overcompensatory density-dependence is increasingly being acknowledged (Zipkin et al. 2009).

GPM suppression strategies are particularly effective against small pest populations, with efficacy increasing asymptotically with decreasing pest density (Klassen 2005). This includes small, isolated pockets of a pest at relatively high density or low densities of a pest dispersed over a wider area (Lance and McInnis 2005). This attribute derives from the fact that GPM strategies suppress populations through decreasing the proportion of viable matings, creating an Allee effect (Tobin et al. 2011). The proportion of non-viable matings increases with an increasing ratio of transgenic:wild individuals (the overflooding ratio), which itself increases as the target population declines. This principle was established theoretically by Knipling (Knipling 1955) and later extended using more sophisticated population models/parameters (reviewed in Ito and Yamamura 2005). A clear example of this dynamic can be seen in the results of the tsetse fly (*Glossina austeni*) SIT eradication programme on the island of Unguja, Zanzibar (Vreysen 2005). Over the course of the programme the overflooding ratio increased from 10:1 to over 100:1 as the population declined, leading to increases in the proportion of non-viable matings (indicated by sterility of collected egg masses). Similarly, during the suppression of *Ae. aegypti* populations on Grand Cayman using RIDL males, the fluorescence ratio (an indication of the number of non-viable matings Harris et al. 2011) increased with the overflooding ratio, both of which corresponded with significant suppression of the target population (Harris et al. 2012). A related, independent advantage of GPM strategies is that transgenic individuals will actively disperse and seek out reproductive partners, making them uniquely efficient at targeting pests that are difficult and/or costly to reach using conventional means, for example where populations are inaccessible or cryptic (Suckling 2003). This is an advantage in inhabited areas or where there are multiple landowners, scenarios that have previously challenged or prevented eradication efforts (Glen et al. 2013; Suckling 2003).

The density-dependent efficiency of GPM strategies makes them highly suitable as ‘end-game’ eradication tactics, driving invasive populations to elimination once numbers have been reduced using conventional tools (Suckling et al. 2012) or potentially after natural population collapses (Simberloff and Gibbons 2004). Facilitating this integrated approach, self-limiting population suppression strategies are predicted to be highly compatible with conventional control methods. For example, female-specific systems display considerable synergy with pesticides (Harvey-Samuel 2015; Alphey et al. 2009), physical removal (e.g. hunting, fishing or trapping) (Thresher 2007) and classical biocontrol (Thresher et al. 2014). The combination of sterile male goat releases (analogous to a bisex-lethal system) and targeted hunting is predicted to decrease the hunting pressure (by c. 40%) and release ratio (by c. 93%) required to control invasive goat populations compared to either method used separately (da Silva et al. 2010).

If the target pest population exhibits strong negative density-dependence, suppression may become increasingly difficult as the population declines, due to the increased survival and/or fitness of remaining individuals (Alphey and Bonsall 2014). Under situations of overcompensatory density-dependence, insufficient releases/density of transgenic individuals could inadvertently increase pest density and/or adult body size (Yakob et al. 2008; Stone 2013; Yakob and Bonsall 2009). This sensitivity to density-dependence is a major factor influencing the efficiency of GPM suppression programmes (Bax and Thresher 2009). Engineering control phenotypes to maintain elements of density-dependent population regulation can help reduce this sensitivity. For example, larval populations of *Ae. aegypti* can exhibit negative density-dependent regulation by intraspecific competition for food resources (reviewed in Legros et al. 2009). RIDL lines targeting *Ae. aegypti* have circumvented this concern by designing transgenes that act after the stage of competition (post-larva). These include late-acting bisex-lethality which causes death at the pupal stage (Phuc et al. 2007) and female-specific systems which allow transgenic females to survive to adulthood but prevent them from flying (and therefore reproducing) (Fu et al. 2010). Theoretical analysis of the late-acting bisex-lethal system suggests that delaying lethality can significantly reduce the overflooding ratios and time-frames required to suppress *Ae. aegypti* populations (Phuc et al. 2007; Yakob and Bonsall 2009) whilst reducing the potential for overcompensatory responses to suppression (Yakob et al. 2008).

### The prevention of re-invasion

Immigration of a pest into a target area can preclude eradication or make its benefits transitory (Myers et al. 2000; Whitten and Mahon 2005). GPM programmes are adept at dealing with immigration due to their ability to be deployed in large-scale barrier zones—an operational strategy where low levels of released individuals create a mating barrier between pest free and infested areas (Hendrichs 2005). Temporary barrier zones are integral to the ‘rolling-carpet’ and ‘wave’ SIT deployment strategies where they may be deployed in front of and/or behind an eradication front to delimit target subpopulations for eradication (Yakob and Bonsall 2009), or prevent long-distance dispersal of pests reinvading previously eradicated areas, respectively. Such a temporary barrier zone was created along 15,000 km^2^ of the east, south and west borders of north-western Libya during the successful 1991 screwworm eradication programme (Lindquist and Abusowa 1992). Barrier zones can also be deployed as more permanent biosecurity measures (Suckling 2003). Generally, these have been created to prevent reinvasion of a pest into an area from which it has been previously eradicated—for example, since 1982 the release of up to 2 billion sterile medfly males per week along the Mexico–Guatemala border has protected the United States, Mexico, Belize and an increasing proportion of Guatemala from reinvasion (Enkerlin et al. 2015). Similarly, barrier zones could be used to prevent invasion in the first instance, by releasing GPM individuals into high-risk transport areas such as airports or docks without the risk of these individuals establishing (Thresher et al. 2014; Lee et al. 2013). Models representing island goat invasions suggest that the presence of as few as 20 sterile (bisex-lethal) males could provide such an effective “sentry system” against future invasion (da Silva et al. 2010).

In vector populations where a gene-drive has spread a disease-refractory transgene to fixation, the subsequent transgenic population would be buffered against reinvasion of vectors carrying that disease, assuming this immigration does not push the transgene below its invasion threshold.

### Resistance prevention

Potential mechanisms for the development of resistance to GPM strategies have been reviewed (Bull 2015; Leftwich et al. 2016) and we limit discussion here to specific examples. GPM resistance mechanisms may be *ecological*—where a heritable change in target population behaviour reduces the proportion of matings achieved by transgenics, or *molecular*—where a genetic change impairs the ability of a transgene to exert a control phenotype. As all pest control methods are susceptible to resistance, the avenues by which these effects are mitigated will determine their suitability to an eradication programme.

Two potential cases of ecological resistance to GPM strategies have been reported, both in SIT programmes targeting tephritids and both involving the ability of wild females to discriminate against released males (assortative mating) (Hibino and Iwahashi 1991; McInnis et al. 1996). While this shows that ecological resistance to GPM strategies is possible, the relative paucity of examples suggests that it is potentially a rare phenomenon. Population modelling suggests that, in some cases, this resistance may be overcome simply by increasing the overflooding ratio, a tactic which resulted in the eradication of a ‘behaviourally resistant’ population of melon fly on Okinawa (Ito 2003). An established management method for ecological resistance is to avoid selecting for discriminable traits in release strains (Leftwich et al. 2016)—for example selection for shorter development periods during artificial rearing was shown to act pleiotropically on mating period, leading to partial reproductive isolation of melon fly lab strains (Miyatake and Shimizu 1999). For some traits, regular introgression of wild and release strains may help retain compatibility, however, this genetic variation can be lost quickly under laboratory rearing (Zygouridis et al. 2014). For plastic behaviours with an environmental component, rearing conditions could be adapted to resemble more natural habitats. For example, domesticated rats reared in outdoor enclosures display increased levels of social aggressiveness and more readily form dominant-subordinate relationships (both characteristics typical of wild rats) (Price 1999).

Research into ecological resistance will be particularly important in evaluating underdominance-based gene-drives as these systems rely on setting up genetically incompatible but interbreeding populations of pests in the field—conditions under which the evolution of reproductive isolation may be predicted (Noor et al. 2001). For homing-based drives, modelling suggests that alleles which allow inbreeding through self-fertilisation may evolve in response to population suppression (Bull 2017). The selective advantage of this adaptation is largely determined by the fitness of offspring from selfed individuals: selfing does not evolve if inbreeding depression is too great. These models applied specifically to hermaphroditic species such as weeds, and so may be less relevant to current GPM targets which are exclusively sexually reproductive. However, an analogous scenario in GPM targets would be the selection of alleles associated with an increased frequency of sibling mating, although this may be more constrained than for systems where higher inbreeding coefficients are possible (Bull 2016).

Molecular resistance has not yet been recorded in GPM programmes, despite experiments explicitly designed to test for its evolution (Gong et al. 2005). The potential consequences of molecular resistance have been theoretically explored for bisex and female-specific lethal systems in insects using models of population genetics and dynamics (Alphey et al. 2011). A key finding was that these tactics display a level of built-in resistance management as released individuals will continually introgress susceptibility alleles into a target population. A similar but orthogonal approach comes from combining GPM releases with a pesticide which does not target the released life-stage. Theoretical (Alphey et al. 2007) and empirical (Harvey-Samuel 2015) studies have shown that female-lethal systems can delay or prevent evolution of resistance to transgenic *Bacillus thuringiensis* (*Bt*) crops by introgression of susceptibility alleles into target pest populations. Similarly, a combination of SIT targeting the Pink Bollworm and *Bt* transgenic cotton was used to suppress the development of *Bt* resistance in Arizona, USA (Tabashnik et al. 2010). The combination of these control tactics is likely to have a similarly beneficial effect on limiting the spread of a hypothetical molecular resistance allele.

Such a ‘combination therapy’ approach can also be taken at the molecular level. A theoretical example involves the use of two or more ‘stacked’ orthogonal, lethal systems to create a “redundant killing” suppression transgene (Eckermann 2014). Similarly, for homing-based drives, mutations introduced into the target cleavage site by the NHEJ repair pathway may create resistance alleles in the target population which the drive transgene will no longer be able to recognise. The release of drive transgenes with multiple sgRNAs designed to target independent points at a target locus would substantially reduce the frequency of these resistance alleles, as discussed previously for HEGs (Deredec et al. 2008). Resistance management could be further enhanced through employing Cas9 variants which cleave DNA at a predictable distance away from their sgRNA recognition site (Zetsche et al. 2015) and through linking the drive to genes engineered to knockdown components of the NHEJ repair pathway (Basu et al. 2015).

Finally, population ecology could be combined with molecular designs to maximise suppression and minimise resistance evolution. For example, inducible GPM strategies (Schliekelman and Gould 2000)—where a deleterious transgene is spread through a target population and activated once it has reached a high frequency—could benefit significantly from the Allee effect. Inducible systems are hypothetical but could be based on the spread of an insect neuropeptide gene which prevented entry into or exit out of diapause (Zhang et al. 2011). If such an “ecological suicide” (Zhang et al. 2011) transgene had spread to high frequency prior to activation, surviving individuals would be at low density and potentially highly dispersed, making them susceptible to Allee effects. Timing lethality to coincide with, or precede, hazardous conditions (e.g. winter as with a diapause preventing neuropeptide) could further magnify these effects as the small, residual, resistant population would show increased susceptibility to environmental stochasticity.

### Transferability

The capacity to deploy a specific technology against multiple pests helps spread development costs and facilitates rapid application against emerging invasives. A dual advantage of GPM strategies is that they are extremely species-specific (relying on mating to affect target populations) but also relatively transferable (Alphey 2002). This is evidenced by single GPM transgenes functioning in multiple species without modification (Supplementary Table 1: ‘Entire transgene’). Examples are primarily in insects, where transfers have taken place at all levels of classification up to and including between families. In vertebrates (fish) there is also evidence of construct transfer at the family level. Further evidence for transferability comes from successful transgene transfer from one species to another after the replacement of species-specific regulatory elements in the transgene construct (Supplementary Table 1: ‘Homologous components’). Again, this has taken place in insects across relatively large phylogenetic distances (up to the family level) and has occurred more frequently in vertebrates than for the transfer of entire constructs.

A broader view of transferability can be gained by exploring the variety of taxa in which particular strategies have been demonstrated (Supplementary Table 2). Of the strategies shown, the most widely transferred are the simplest, with bisex-lethal and female-lethal GPM strategies demonstrated in a diverse range of taxa. Self-sustaining strategies have been demonstrated in fewer target species. However, CRISPR/Cas9 based homing-drives have been recently demonstrated in organisms as distant as yeast and mosquitoes hinting at the versatile nature of this genome editing technology.

## Conclusion

For many invasive species which threaten biodiversity, there is no cost-efficient or sufficiently effective means of control (Thresher et al. 2014). Transgene-based GPM strategies represent a next generation approach which may be of use against these intractable pests. These technologies have been successfully transferred to a variety of pest insects and vertebrates and display characteristics which may make them highly suitable for integration into the current toolset for eradicating invasive species. Uniquely, they also possess the flexibility, under some circumstances, to replace an invader with a more benign form, rather than remove it from a target ecosystem. However, challenges to the implementation of these technologies remain. Particularly in the case of self-sustaining gene-drives, concerns surrounding the risk of transgene escape predominate. The rapid emergence of multiple, independent designs for safeguards, however, shows the seriousness with which these concerns are taken. We agree with others in acknowledging the risks associated with these technologies and argue that these risks can only be properly assessed and safeguard designs evaluated given functioning systems and a willingness to test these systems under sufficiently controlled, realistic conditions (Oye and Esvelt 2014; National Academies of Sciences, Engineering and Medicine 2016). Preliminary frameworks for the assessment and management of risk associated with these controlled field experiments exist (Oye et al. 2014; Benedict et al. 2008) and continue to evolve (National Academies of Sciences, Engineering and Medicine 2016). In the case of invasive species, the geographic isolation, low carrying capacities, high endemism and invasion susceptibility of island ecosystems makes them both ideal locations for these trials and attractive GPM targets.

### Box 1: Disease-refractory transgenes

Population replacement strategies aim to spread ‘disease-refractory’ transgenes which reduce the ability of vectors inheriting them to transmit disease. Research has primarily focused on identifying sufficiently active ‘effector molecules’ which, when expressed in appropriate tissues within the vector, effectively reduce disease transmission rates (Wang and Jacobs-Lorena 2013).

An example of conservation value would be the development of refractory transgenes targeting the avian malaria parasite *P. relictum*. Transmission of *P. relictum* by the invasive mosquito *C. quinquefasciatus* has contributed to the extinction of multiple endemic forest bird species in Hawaii and poses a threat to other naïve island avian communities (LaPointe et al. 2012). A *P. relictum* refractory engineered mosquito has not yet been demonstrated, however, research into refractory transgenes targeting the closely related avian malaria parasite *P. gallinaceum* has taken place. Transgenic *Ae. aegypti* mosquitoes engineered to overexpress two endogenous antimicrobial peptides -AMPs-(Cecropin A and Defensin A) were found to be unable to transmit *P. gallinaceum* to chickens (Kokoza et al. 2010). The broad-spectrum effects of AMPs suggests that they may be useful in designing refractory transgenes targeting *P. relictum* and other Plasmodium spp. Additionally, single-chain antibodies (scFvs) targeting *Plasmodium* chitinase protein have been shown to significantly reduce *P. gallinaceum* transmission to *Ae. aegypti* through infected bloodmeals (Li et al. 2005) and the *P. relictum* chitinase genes have been identified (Garcia-Longoria 2014). Transgenic *Ae. aegypti* engineered to express scFvs targeting *P. gallinaceum* circumsporozoite protein were still, however, able to transmit *P. gallinaceum* despite having substantially reduced numbers of sporozoites in their salivary glands (Jasinskiene et al. 2007). With germ-line transformation of *C. quinquefasciatus* achieved (Allen et al. 2001), the testing of these and other potentially refractory transgenes in an ecologically relevant system is possible.

### Box 2: Case studies

#### Insects

*Philornis downsi*, a fly native to mainland South America, is invasive in the Galapagos Islands. Adults lay eggs in birds’ nests where larvae are obligate ectoparasites of developing nestlings (Fessl and Tebbich 2002). *P. downsi* threatens many native Galapagos land birds and is the primary cause of decline in the critically endangered mangrove finch *Camarhynchus heliobates* (O’Connor et al. 2010). Currently, there are no effective control measures (Causton et al. 2013). Information regarding mating behavior is limited, however, other biological characteristics—benign adult life-stages and a relatively short life-cycle—would be advantageous in targeting *P. downsi* using genetic pest management (GPM). The existence of an artificial larval diet (Nichols and Ulrich 2014) and lack of strong mating barriers within its invaded range (Dudaniec et al. 2008) are also positive. Transgenesis of the *Philornis* genus has not been reported, however, germ-line transformation of the closely related housefly *Musca domestica* has been achieved (Hediger et al. 2001). Several female-lethal systems have been constructed in dipterans using the sex-alternative splicing behaviour of the *transformer* (*tra*) gene (Fu et al. 2007; Scott 2014). *tra* appears to display functional conservation at the family level in Schizophorid dipterans: female-lethal transgenes constructed using either Mediterranean fruitfly or New World screwworm *tra* functioned in other members of the Tephritidae and Calliphoridae, respectively (Scott 2014). Encouragingly, both these constructs also functioned in the more distantly related *D. melanogaster*. The characterized *M. domestica tra* gene (Hediger et al. 2010) may therefore be of use in constructing an fsRIDL transgene targeting *P. downsi*.

#### Mammals

Three species of rat (black rat *Rattus rattus*, Polynesian rat *Rattus exulans* and Norway rat *Rattus norvegicus*) are amongst the most damaging invasives of island ecosystems (Towns et al. 2006). Rats and mice represent the most promising vertebrate targets for GPM strategies: they have short generation times/life-spans; they are easily and cheaply reared in large numbers; extensive literature exists on their mating systems, invasion ecology and population biology (Harper and Bunbury 2015), including details relevant to GPM programmes (Gould 2008) and germ-line transformation and conditional (including tetracycline-repressible) gene expression systems are available (Schonig 2012; Lewandoski 2001). Development of bisex-lethal transgenes in rats would benefit from preliminary research into engineering these technologies in mice (Thresher et al. 2000). Encouraging development of female-specific strategies: in both mice and rats, transgenic lines have been created which show sex-specific expression of reporter genes (Hinshelwood et al. 2000; Li et al. 2014). Modelling indicates that large, established rat populations could be eradicated in less than 20 years using female-specific GPM strategies. This timeframe would diminish substantially if combined with existing control methods (Thresher 2007).

An alternative method under investigation in mice is to co-opt the naturally occurring ‘*t*-haplotype’ selfish-genetic element as the basis for a self-sustaining Sex Ratio Distortion (SRD) system (D. Threadgill: personal communication). The *t*-haplotype is an autosomal gene complex found on mouse chromosome 17 consisting of a number of ‘distorter’ loci which impair sperm motility and a ‘responder’ locus which rescues motility in *t*-haplotype^+^ sperm (Herrmann and Bauer 2012). Thus in heterozygous males, transmission of the *t*-haplotype can reach upwards of 90%. Transgenes carrying t-haplotype elements have shown comparable rates of transmission distortion (>95% of progeny inherit the transgene) in both laboratory and island population mouse genetic backgrounds (D. Threadgill: personal communication). Currently, these transgenes are being engineered to include the *SRY* gene—a male determining locus previously shown to cause female-to-male sex reversal in transgenic mice (Koopman et al. 1991) to create a self-sustaining SRD system (D. Threadgill: personal communication). The *t*-haplotype is found worldwide but at lower frequencies (10–25%) than predicted by its driving nature (Ardlie and Silver 1996). Proposed mechanisms for this “*t*-haploype paradox” include sexual selection effects, which might be pre-copulatory (females able to discriminate against *t*-haplotype males Manser et al. 2015) or post-copulatory (*t*-haplotype males produce less competitive sperm Sutter and Lindholm 2015). Although the proposed SRD transgene would likely drive more aggressively than the endogenous *t*-haplotype complex (as the synthetic drive system would only function in and only produce males) a better understanding of these naturally occurring resistance mechanisms would help to predict potential field performance of this system.

#### Reptiles

The brown tree snake (*B. irregularis*) was introduced to the island of Guam shortly after WWII, rapidly causing the extirpation of over 80% of Guam’s forest bird species and a number of native bats and lizards. Persistence of the snake on Guam precludes re-establishment of avian communities and provides a source population for invasion to other naïve islands (Rodda and Savidge 2007). Brown tree snakes on Guam occur at high densities and perhaps due to a lack of sufficient prey it appears that many adults are not reproductively active (Moore et al. 2005). This would prove advantageous in deploying a population suppression strategy as the overflooding ratio (number of released snakes required to cause suppression) would be substantially lower than population density would suggest. However, to avoid an overcompensatory response to suppression as competition for food is relaxed (Campbell 1996) a late-acting bisex or female-specific/SRD strategy may be most appropriate. Although information regarding life-history and basic mating biology in the wild is limited, protocols for lab rearing are available (Greene et al. 1997). Genetic engineering of snakes (including Colubrids) has been reported, although it is unclear whether this extends to germ-line transformation (Mozdziak and Petitte 2006). Female-lethal/daughterless strategies utilizing the *vitellogenin*-*1* promoter or targeting aromatase are functional in fish and this could serve as a framework for their development in snakes. The Burmese python genome has been sequenced and putative *vitellogenin* and *aromatas*e homologs identified.

The red-sided garter snake (*Thamnophis sirtalis parietalis*) is a better-known laboratory model in the same family as the brown tree snake and may serve as a model for developing GPM strategies in this pest. In this species, castration of males induces a “she-male” phenotype: males produced female sex-pheromone and were actively courted by other males (Parker and Mason 2014). This she-male phenotype was also induced in males given estrogen implants (Parker and Mason 2012) but was absent in castrated males given an aromatase inhibitor (Parker and Mason 2010). This suggests a role for aromatase in inducing female mating behaviour in this species. Future experiments could examine whether females given aromatase blockers prior to reproductive development show reduced female-pheromone activity, or display male reproductive physiology/behavior. With preliminary trials of aerial bait delivery suggesting significant success at reducing brown tree snake activity (Clark and Savarie 2012), eradication of this species may be achievable. A GPM strategy could function effectively as an end-game eradication tactic if used sequentially alongside this control method.

## Electronic supplementary material

Below is the link to the electronic supplementary material.

Supplementary material 1 (DOCX 51 kb)
