# Supplementary material for: Towards the genetic control of invasive species
Source: Biol Invasions. 2017 Feb 21;19(6):1683–703. doi: 10.1007/s10530-017-1384-6 (PMC5446844; doi:10.1007/s10530-017-1384-6)
Supplement: Supplementary file 1 — Supplementary material 1 (DOCX 51 kb) [file 10530_2017_1384_MOESM1_ESM.docx]

**Supplementary Information**

Supplementary Table 1: Published evidence for transfer of GPM transgenes between species. Transfer types include where unmodified constructs have been used in multiple species (‘Entire construct’) and where constructs have been transferred after inclusion of regulatory sequences from their target species (‘Homologous components’). Transfer level states the highest taxonomic level that the transfer has taken place at i.e. ‘family’ refers to constructs moved between different families in the same order, ‘genus’ between different genera within the same family and ‘species’ between different species within the same genus.

| Hierarchy of transfer | Evidence of transferability | Transfer level |
| --- | --- | --- |
| Entire construct | fsRIDL construct OX4319 using *Bombyx mori dsx* intron functional in lepidopterans – *Pectinophora gossypiella*, *Plutella xylostella* [[1](#_ENREF_1)] and *Bombyx mor*i [[2](#_ENREF_2)] | family |
|  | fsRIDL construct OX3907 using *Ceratitis capitata tra* intron functional in tephritids – *Ceratitis capitata [*[*3*](#_ENREF_3)*], Anastrepha ludens* [[4](#_ENREF_4)] and *Bactrocera oleae* [[5](#_ENREF_5)] | genus |
|  | fsRIDL construct OX3077 using *Ceratitis capitata tra* intron functional in tephritids – *Ceratitis capitata* and *Drosophila melanogaster* [[3](#_ENREF_3)] | family |
|  | fsRIDL construct OX3604 using *Aedes aegypti Act-4* promoter functional in *Aedes aegypti* [[6](#_ENREF_6)] and *Aedes albopictus* [[7](#_ENREF_7)] | species |
|  | fsRIDL construct OX4358 using *Aedes albopictus* *Act-4* promoter functional in *Aedes aegypti* and *Aedes albopictus [*[*7*](#_ENREF_7)*]* | species |
|  | fsRIDL construct FL11 using *Lucillia cuprina tra* functional in both *Lucilia cuprina* and *Cochliomyia homnivorax* [[8](#_ENREF_8), [9](#_ENREF_9)] | genus |
|  | two-part fsRIDL constructs using *Ceratits capitata tra* and *Anastrepha suspensa* embryonic *As-sry-α* promoter functionality in *Anastrepha ludens* and *Anastrepha suspensa* [[10](#_ENREF_10), [11](#_ENREF_11)] | species |
|  | bisex-lethal construct using zebrafish *zSMAD5* promoter to control expression of zebrafish developmental gene (*zBMP2*). Functional in zebrafish and catfish [[12](#_ENREF_12)] | family |
| Homologous components | *Act-4* promoter from *Aedes aegypti* replaced with homolog from *Aedes stephensi.* Female-specific construct functional in *Aedes stephensi* [[13](#_ENREF_13)] | genus |
|  | Analogous design to [[10](#_ENREF_10)] built in *Lucillia cuprina* using *Lucillia sericata* *Lsbnk* promoter and *Lucillia sericata* proapoptotic gene (*Lshid*)[[14](#_ENREF_14)] | family |
|  | fsRIDL construct FL3 transferred from *Drosophila melanogaster* to *Lucillia cuprina* after replacement of *Drosophila melanogaster* *hsp70* promoter with *lucillia cuprina* homolog [[9](#_ENREF_9)] | family |
|  | Embryonic bisex-lethal construct [[15](#_ENREF_15)] transferred from *Drosophila melanogaster* to *Ceratitis capitata* after replacement of *Drosophila melanogaster* *sry-α* promoter with *Ceratitis capitata* homolog [16](#_ENREF_16)] | family |
|  | Female-lethal construct transferred from zebrafish to common carp after replacement of zebrafish *vtg1* promoter with common carp homolog [[17](#_ENREF_17)] | genus |
|  | SRD construct transferred from medaka to zebrafish after replacement of zebrafish ovarian aromatase blocker with medaka homolog [[18](#_ENREF_18)] | family |

Supplementary Table 2: Published evidence of the different classifications in which the GPM strategies discussed in this review have been demonstrated. Where different molecular routs have been taken to achieve the same strategy in a taxanomic group these are listed (Mechanism) with the first published example of each mechanism in that group cited. Not all these examples are discussed in this review.

| Strategy | Demonstrated in | Mechanism |
| --- | --- | --- |
| Bisex-lethal | Diptera | tTAV lethal positive feedback loop [[19](#_ENREF_19)], tet-off system + lethal effector [[15](#_ENREF_15)] |
|  | Lepidoptera | tTAV lethal positive feedback loop [[20](#_ENREF_20)] |
|  | Mollusc | tet-off system + lethal effector [[12](#_ENREF_12)] |
|  | Fish | tet-off system [[12](#_ENREF_12)], copper sulfate, cadmium sulfate and sodium chloride repressible promoter + lethal effector [[21](#_ENREF_21), [22](#_ENREF_22)] |
|  | Plants | Genetic use Restriction Technologies (GURTs) [[23](#_ENREF_23)], Recoverable Block of Function [[24](#_ENREF_24)] |
| Female-specific lethal | Diptera | sex-alternate splicing [[3](#_ENREF_3)], sex specific promoters [[25](#_ENREF_25)] |
|  | Lepidoptera | sex-alternate splicing [[1](#_ENREF_1)] |
|  | Fish | sex-specific promoter [[17](#_ENREF_17)] |
| SRD | Diptera | HEG targeting X chromosome [[26](#_ENREF_26)] |
|  | Fish | Aromatase blocker [[18](#_ENREF_18)] |
| Underdominance drive | Diptera | CRP gene [[27](#_ENREF_27)], MEDEA element [[28](#_ENREF_28)] |
| Homing-drive | Diptera | HEG [[29](#_ENREF_29)], TALEN & ZFN [[30](#_ENREF_30)], CRISPR/Cas9 [[31](#_ENREF_31)] |
|  | Yeast | CRISPR/Cas9 [[32](#_ENREF_32)] |

**References**

1 Jin, L.*, et al.* (2013) Engineered Female-Specific Lethality for Control of Pest Lepidoptera. *ACS Synthetic Biology* 2, 160-166

2 Tan, A.*, et al.* (2013) Transgene-based, female-specific lethality system for genetic sexing of the silkworm, Bombyx mori. *Proceedings of the National Academy of Sciences of the United States of America* 110, 6766-6770

3 Fu, G.*, et al.* (2007) Female-specific insect lethality engineered using alternative splicing. *Nature Biotechnology* 25, 353-357

4 Morrison, N.I.*, et al.* (2010) Genetic improvements to the sterile insect technique for agricultural pests. *Asia Pacific Jounral of Molecular Biology and Biotechnology* 18, 275-295

5 Ant, T.*, et al.* (2012) Control of the olive fruit fly using genetics-enhanced sterile insect technique. *BMC Biology* 10, 51-51

6 Fu, G.L.*, et al.* (2010) Female-specific flightless phenotype for mosquito control. *Proceedings of the National Academy of Sciences of the United States of America* 107, 4550-4554

7 Labbe, G.M.C.*, et al.* (2012) Female-Specific Flightless (fsRIDL) Phenotype for Control of Aedes albopictus. *Plos Neglected Tropical Diseases* 6

8 Concha, C.*, et al.* (2016) A transgenic male-only strain of the New World screwworm for an improved control program using the sterile insect technique. *BMC Biology* 14

9 Li, F.*, et al.* (2014) Transgenic sexing system for genetic control of the Australian sheep blow fly Lucilia cuprina. *Insect Biochemistry and Molecular Biology* 51, 80-88

10 Schetelig, M.F. and Handler, A.M. (2012) A transgenic embryonic sexing system for Anastrepha suspensa (Diptera: Tephritidae). *Insect Biochemistry and Molecular Biology* 42, 790-795

11 Schetelig, M.F.*, et al.* (2016) Tetracycline-suppressible female lethality and sterility in the Mexican fruit fly, Anastrepha ludens. *Insect Molecular Biology* 25, 500-508

12 Thresher, R.*, et al.* (2009) Development of repressible sterility to prevent the establishment of feral populations of exotic and genetically modified animals. *Aquaculture* 290, 104-109

13 Marinotti, O.*, et al.* (2013) Development of a population suppression strain of the human malaria vector mosquito, Anopheles stephensi. *Malaria Journal* 12, 142

14 Yan, Y. and Scott, M.J. (2015) A transgenic embryonic sexing system for the Australian sheep blow fly Lucilia cuprina. *Scientific Reports* 5

15 Horn, C. and Wimmer, E.A. (2003) A transgene-based, embryo-specific lethality system for insect pest management. *Nature Biotechnology* 21, 64-70

16 Schetelig, M.F.*, et al.* (2009) Conditional embryonic lethality to improve the sterile insect technique in Ceratitis capitata (Diptera: Tephritidae). *BMC Biology* 7

17 Thresher, R.*, et al.* (2014) Sex-ratio-biasing constructs for the control of invasive lower vertebrates. *Nature Biotechnology* 32, 424-427

18 Thresher, R.E.*, et al.* (2005) Genetic control of sex ratio in animal populations. *Australian Patent 782109*

19 Gong, P.*, et al.* (2005) A dominant lethal genetic system for autocidal control of the Mediterranean fruitfly. *Nature Biotechnology* 23, 453-456

20 Morrison, N.I.*, et al.* (2012) Engineered Repressible Lethality for Controlling the Pink Bollworm, a Lepidopteran Pest of Cotton. *Plos One* 7

21 Su, B.*, et al.* (2015) Suppression and restoration of primordial germ cell marker gene expression in channel catfish, Ictalurus punctatus, using knockdown constructs regulated by copper transport protein gene promoters: Potential for reversible transgenic sterilization. *Theriogenology* 84, 1499-1512

22 Su, B.F.*, et al.* (2015) Effects of transgenic sterilization constructs and their repressor compounds on hatch, developmental rate and early survival of electroporated channel catfish embryos and fry. *Transgenic Research* 24, 333-352

23 Lombardo, L. (2014) Genetic use restriction technologies: a review. *Plant Biotechnology Journal* 12, 995-1005

24 Kuvshinov, V.*, et al.* (2001) Molecular control of transgene escape from genetically modified plants. *Plant Science* 160, 517-522

25 Thomas, D.D.*, et al.* (2000) Insect population control using a dominant, repressible, lethal genetic system. *Science* 287, 2474-2476

26 Galizi, R.*, et al.* (2014) A synthetic sex ratio distortion system for the control of the human malaria mosquito. *Nature Communications* 5, 3977-3977

27 Reeves, R.G.*, et al.* (2014) First Steps towards Underdominant Genetic Transformation of Insect Populations. *Plos One* 9

28 Akbari, O.S.*, et al.* (2013) A Synthetic Gene Drive System for Local, Reversible Modification and Suppression of Insect Populations. *Current Biology* 23, 671-677

29 Windbichler, N.*, et al.* (2011) A synthetic homing endonuclease-based gene drive system in the human malaria mosquito. *Nature* 473, 212-215

30 Simoni, A.*, et al.* (2014) Development of synthetic selfish elements based on modular nucleases in Drosophila melanogaster. *Nucleic Acids Research* 42, 7461-7472

31 Gantz, V.M. and Bier, E. (2015) The mutagenic chain reaction: A method for converting heterozygous to homozygous mutations. *Science* 348, 442-444

32 DiCarlo, J.E.*, et al.* (2015) Safeguarding CRISPR-Cas9 gene drives in yeast. *Nature Biotechnology* 33, 1250-1255
